# The Impact of COVID-19 Pandemic on Individuals With Pre-existing Obsessive-Compulsive Disorder in the State of Qatar: An Exploratory Cross-Sectional Study

**DOI:** 10.3389/fpsyt.2022.833394

**Published:** 2022-04-12

**Authors:** Maryam Siddiqui, Ovais Wadoo, James Currie, Majid Alabdulla, Areej Al Siaghy, Abdulkarim AlSiddiqi, Eslam Khalaf, Prem Chandra, Shuja Reagu

**Affiliations:** ^1^Hamad Medical Corporation, Doha, Qatar; ^2^Qatar University, Doha, Qatar; ^3^Weill Cornell Medicine-Qatar, Ar-Rayyan, Qatar

**Keywords:** Qatar, Obsessive-Compulsive Disorder (OCD), COVID-19 pandemic, Yale-Brown Obsessive-Compulsive Scale (YBOCS), OCD subtypes

## Abstract

**Background:**

Published evidence about the impact of the COVID-19 pandemic on Obsessive-Compulsive Disorder (OCD) is conflicting. Most studies suggest an increase in the severity of OCD in people with pre-existing OCD, whereas some do not.

**Aim:**

Given the conflicting evidence globally and lack of data from the Arab world, we aimed to explore the impact of the pandemic on obsessive-compulsive symptoms in adults with pre-existing OCD.

**Methods:**

A telephonic questionnaire-based cross-sectional study among adults with pre-existing OCD and specifically with fear of contamination and washing compulsions being major symptom dimensions of OCD. The severity of OCD during the pandemic was compared with their pre-pandemic scores. The severity of OCD was assessed using the Yale-Brown Obsessive-Compulsive Scale (YBOCS).

**Results:**

Those with the duration of diagnosis of OCD of<10 years showed a statistically significant increase in the mean YBOCS score of 5.54 from pre-pandemic to during pandemic, which was significant at *p* = 0.006. This significance was maintained across the Compulsive and Obsessive subsets of the scale.

**Conclusion:**

Adults with pre-existing OCD with fear of contamination reported a statistically significant increase in severity of obsessive-compulsive symptoms only if the duration of their OCD diagnosis was relatively shorter (<10 years). In the context of the conflicting evidence regarding the worsening of OCD symptoms due to the unique infection control measures of this pandemic, this study highlights the importance of the impact of the duration of the disorder and the subtype of the disorder. Such classification might help public health resources to be directed better at those most at risk and also help us understand the very nature of this disorder better.

## Introduction

Coronavirus Disease 2019 was declared as a public health emergency by the WHO in March 2020 (1). This public health emergency prompted large-scale lockdown to limit the spread of infection and various public health measures were implemented including frequent handwashing, use of face masks, social distancing, and quarantining of infected individuals (2, 3). Infectious epidemics are associated with an increased level of anxiety and stress in the healthy population and worsen symptoms among individuals with pre-existing mental disorders (4, 5). The vulnerability of individuals with pre-existing mental disorders during the COVID-19 outbreak has been widely reported in scientific publications and Obsessive-Compulsive Disorder (OCD) is particularly relevant because of the widespread fear of contamination and ritualized cleaning behavior advocated to limit the spread of infection (6–8). Similar findings have been reported from previous outbreaks such as Severe Acute Respiratory Syndrome (SARS) and novel influenza (H1N1), where obsessive-compulsive symptoms worsened due to fears of acquiring infection (9, 10). In the current pandemic, some commentators have considered patients with OCD to be particularly vulnerable, especially patients with obsessions around contamination (11). It was thus speculated at the start of the pandemic that the OCD symptoms will increase during the pandemic. However, the published evidence about the impact of the pandemic on OCD so far is conflicting. Multiple studies in nonclinical samples have reported an increase in OCD symptoms (12–14). Most of the studies suggest an increase in OCD in people with manifest OCD during the pandemic (15–19). Whereas some do not (20).

In an online survey conducted in November 2020 in Germany, it was found that the majority of participants with OCD were negatively affected by the COVID-19 pandemic, and the negative effects were more pronounced in people with hand washing compulsions. A total of 72% of the participants reported an increase in OCD. This increase was significantly stronger in washers (15, 21). Ji G et al. conducted a prospective cohort survey study in China (21). It examined if fear of negative events affects Yale-Brown Obsessive-Compulsive Scale (Y-BOCS) scores in the context of a COVID-19–fear-invoking environment. It was concluded that the findings indicate that the fear of COVID-19 was associated with a greater Y-BOCS score, suggesting that an environment (COVID-19 pandemic) × psychology (fear and/or anxiety) interaction might be involved in OCD and that fear of negative events might play a role in the etiology of OCD. Data from Italy in July 2020 supports the notion of the exacerbation of OCD symptoms due to the COVID-19 pandemic, as about one-third of their sample reported clinical worsening of OCD (16, 17).

Tanir et al. (18) looked at a sample of children and adolescents with a primary diagnosis of OCD in a university hospital (18). They report a significant increase in the frequency of contamination obsessions and cleaning/washing compulsions during the pandemic period with a significant relationship with talking/searching in the social environment about COVID-19, daily preoccupation about COVID-19, duration of OCD diagnosis, and diagnosis of COVID-19 in someone familiar. Nissen et al. (19) explored the immediate effect of the COVID-19 pandemic on two groups of children and adolescents, one newly diagnosed with OCD and the other, who was diagnosed years ago and their primary treatment was completed. They report worsening of symptoms in both groups but more pronounced in the group who were diagnosed years ago (19).

Chakraborty and Karmakar 2020 did not find any increase in obsessive and compulsive symptoms in patients with an obsession with contamination and compulsive washing before the pandemic. This study conducted in India report a very small proportion of patients (6%) experiencing symptoms of exacerbation (20).

There are limited studies looking at the impact of the pandemic on OCD in the Arab world. Khan et al. explored the association of pandemic with obsessive-compulsive symptoms in adolescents with preexisting mental and behavioral disorders in Qatar and reports significant pandemic fears associated with obsessive-compulsive symptoms (22).

Given the conflicting evidence globally and limited data from the Arab world, we aimed to explore the impact of pandemic fears on obsessive-compulsive symptoms in adults with pre-existing OCD in Qatar. Qatar is a peninsula in the Arabian Gulf, with a population of ~2.7 million, of which ~75% are men, and operates a publicly funded healthcare system (23).

## Methods

### Design, Settings, and Participants

This was a cross-sectional, single-center, exploratory study conducted within the adult mental health service, Hamad Medical Corporation, Qatar. The patients with a pre-existing primary diagnosis of OCD (with fear of contamination and washing compulsions being major symptom dimensions of OCD) were included in the study. The list of participants was obtained through a system-generated search based on the diagnostic code of OCD, which generated a sample of 300 patients registered with adult mental health services. In total, 36 patients out of 300 were identified to have fear of contamination and washing compulsions as a major symptom dimension of their OCD by reviewing their clinical information and thus deemed eligible for the study. At the time of the data collection, in January 2021, the State of Qatar was under a strict lockdown at that time with strict measures to contain the spread of infection. There were restrictions of gatherings, schools were closed, and it was encouraged to work from home.

#### Inclusion Criteria

All adult patients aged 18–65 with a pre-existing primary diagnosis of OCD with fear of contamination and washing compulsions being major symptom dimensions of OCD and agreed to participate were eligible.

#### Exclusion Criteria

Inability to consent for participation in the study due to underlying mental or physical health conditions or inability to engage with the interview.

All eligible patients consented to participate, and all were selected as the number was small. The study design was exploratory, we did not set and formulate any prior statistical hypothesis and, therefore, we did not perform any formal sample size calculation.

### Measures

The survey questionnaire included two sections. The first section was designed to collect data on demographic characteristics (age, gender, and ethnicity) and information regarding the duration of OCD, psychiatric co-morbidity, current medication, and mitigating factors. The second section assessed the severity of OCD symptoms using the Y-BOCS (24–27). The survey questionnaires were in English and Arabic, and the validated Arabic translation was used. The initial draft of the composite data collection tool was piloted on five participants by two researchers to assess the feasibility of the data collection tool. Modifications were made to the data collection tool to account for missing or unclear information and a final version was approved after discussion with the wider team. To achieve maximum reliability among the raters, a training session about the rating methods and terms was carried out.

The Y-BOCS is the gold standard to evaluate the severity of OCD symptoms. It is the most widely used semi-structural scale in both clinical and research settings. Y-BOCS is a validated and established self-administered tool combining self-reported and clinician-rated questions. The Y-BOCS is sensitive to change and hence helps to determine improvements or exacerbation of the disorder symptomatology. Studies of the Y-BOCS have shown that it has adequate psychometric characteristics, including good interrater reliability and predictive validity (28). It consists of a comprehensive symptom checklist to identify the specific type and content of obsessive and compulsive symptoms in addition to a 10-item rating scale. The scale is divided into two subscales that separately measure obsessions and compulsions. For each subscale, five aspects of obsessive and compulsive pathology are each rated on a scale ranging from 0 (no symptoms) to 4 (extreme symptoms): time spent, degree of interference, distress, resistance (greater resistance is assigned lower scores), and perceived control over the symptom. Subscale scores are summed to yield a Y-BOCS total score. A total score of 0–7 is considered nonclinical. Scores ranging between 8 and 15 are considered mild. Scores between 16 and 23 are considered moderate and scores between 24–31 and 32–40 are considered severe and extreme, respectively. These instruments were administered by healthcare professionals, proficient in both English and Arabic languages comprising two psychiatry trainees. Both trainees were proficient in administering the tools. Interviews were carried out in the month of January 2021, and the data was collected for this study.

### Ethical Considerations

The study was reviewed and approved by the Medical Research Center (MRC-01-20-105) and the Institutional Review Board (IRB) at Hamad Medical Corporation, Qatar. Phone calls were made to all participants included in the sample to invite them to the study. Consent of the participating individuals was obtained verbally over the phone and recorded. All information relevant to the study including its purpose, impact on clinical care, and confidentiality safeguards were provided.

#### The Strategy of Data Analysis

All analyses were conducted with IBM SPSS® Statistics version 26 (IBM, NY, USA).

Paired Sample *t*-test was used to compare the means from the same group at different times. This test helped us to know how significant the differences were between the groups.

Pearson correlation test was used to determine the linear correlation between the two sets of data (pre-pandemic and pandemic).

Mann Whitney U test was used to compare differences between two independent groups when the dependent variable is either continuous or ordinal but not normally distributed.

Differences in Obsessions, compulsions, and total YBOCS scores during pre-pandemic and pandemic times were measures.

## Results

### Socio-Demographic Characteristics

Of these 36 patients, about 70% were females compared to 30% males which are in line with the global prevalence of this illness. Qataris constituted 30% of the sample although they represent only around 10% of the population (23). *N* = 15 (41.7%) had a duration of the diagnosis of OCD for over 10 years. *N* = 13 (36%) of the individuals had a co-morbid psychiatric illness, of which the majority, *N* = 10 had depression. Majority, *N* = 30 were maintained on ant-depressants and only *N* = 6 individuals were on anti-psychotics (Table 1).

**Table 1:** Socio-demographic data of the participants a pre-existing primary diagnosis of OCD with fear of contamination and washing compulsions being major symptom dimension of OCD.

**Gender**	**Male**	**Female**							
	**11 (30.65)**	**25 (69.4%)**							
Age	Equal to or less than 35 years	More than 35 years							
	50%	50%							
Nationality	Qatari	Egyptian	Indian	Jordanian	Pakistani	Sudanese	Palestinian	Iranian	Syrian
	30.60%	19.50%	13.90%	13.90%	13.90%	5.60%	5.60%	2.80%	2.80%
Duration of OCD	<10 years	More than 10 years							
	58.30%	41.70%							
Co-morbidities	Depression	Panic Disorder	BPAD	nil					
	27.90%	2.80%	2.80%	63.90%					
Anti-depressants	Fluoxetine	Escitalopram	Sertraline	Clomipramine	Venlafaxine	Paroxetine	Mirtazapine	Fluvoxamine	No anti-depressant
	22.20%	16.70%	11.20%	11.20%	2.80%	2.80%	2.80%	2.80%	27.80%
Anti-psychotics	Olanzapine	Abilify	Amisulpride	Flupenthixol	No Anti-psychotics				
	5.60%	5.60%	2.8%	2.8%	83.30%				

*OCD, Obsessive Compulsive Disorder*.

### Differences Between the Scores of Patients With OCD Before and During COVID-19

The mean YBOCS before the pandemic was 15.6 (SD 9.4) and the mean YBOCS during the pandemic was 18.08 (SD 10.6), with no significant statistical difference for the group as a whole (*p* = 0.11, 2-tailed paired *t*-test) (Table 2).

**Table 2:** Comparison between the pre-pandemic and during pandemic scores for YBOCS for individuals with pre-existing diagnosis of OCD.

	**Obsessions score**	**Compulsions score**	**Total YBOCS**
**Pre-pandemic**
Mean (SD)	8.03 (4.48)	7.61 (5.90)	15.64 (9.45)
**During pandemic**
Mean (SD)	9.42 (5.49)	8.67 (5.62)	18.08 (10.68)
*T*-Test (*p* value)	0.13	0.20	0.10

*YBOCS, Yale-Brown Obsessive-Compulsive Scale*.

*OCD, Obsessive Compulsive Disorder*.

The mean YBOCS before the pandemic for the Obsessions scale was 8.03 (SD 4.84) and the mean YBOCS during the pandemic was 9.42 (SD 5.5), with no significant statistical difference for the group as a whole (*p* = 0.13, 2-tailed paired *t*-test) (Table 2).

The mean YBOC before the pandemic for the Compulsions scale was 7.61 (SD 5.9) and the mean YBOCS during the pandemic was 8.67 (SD 5.6), with no significant statistical difference for the group as a whole (*p* = 0.20, 2-tailed paired *t*-test) (Table 2).

There were no significant correlations between changes in Y-BOCS scores and socio-demographic variables. Those with the duration of diagnosis of OCD of <10 years, *N* = 21, showed a statistically significant increase in the mean YBOCS score of 5.54 from pre-pandemic to during pandemic, which was significant at *p* = 0.006, Tables 3, 4.

**Table 3:** Y-BOCS scores for OCD Duration <10 years.

		**Mean**	**N**	**Standard deviation**	**Standard error mean**
Pair 1	Total YBOCS pre-pandemic	14.52	21	8.346	1.821
	Total YBOCS during pandemic	20.05	21	11.530	2.516
Pair 2	Obsessions score pre-pandemic	7.71	21	4.518	0.986
	Obsessions score during pandemic	10.48	21	6.022	1.314
Pair 3	Compulsions score pre-pandemic	6.81	21	4.611	1.006
	Compulsions score during pandemic	9.57	21	5.938	1.296

*YBOCS, Yale-Brown Obsessive-Compulsive Scale*.

*OCD, Obsessive Compulsive Disorder*.

**Table 4:** Paired Samples Test for changes In Y-BOCS scores for OCD duration <10 years.

		**Mean**	**Std. Deviation**	**Std. Error mean**	**95% confidence interval of the Difference**		**t**	**df**	**Sig. 2-Tailed**
					**Lower**	**Upper**			
Pair 1	Total YBOCS Pre-Pandemic - Total YBOCS During Pandemic	−5.524	8.238	1.798	−9.274	−1.774	−3.073	20	0.006
Pair 2	Obsessions Score Pre-Pandemic - Obsessions Score During Pandemic	−2.762	4.989	1.089	−5.033	−0.491	−2.537	20	0.020
Pair 3	Compulsions Score Pre-Pandemic - Compulsions Score During Pandemic	−2.762	3.936	0.859	−4.553	−0.970	−3.216	20	0.004

*YBOCS, Yale-Brown Obsessive-Compulsive Scale*.

*OCD, Obsessive Compulsive Disorder*.

This difference was maintained even for individual components of the Y-BOCS scale for this group. The Obsessions and the Compulsions subscales of the Y-BOCS showed a significant change in scores of 0.02 and 0.004 respectively, Tables 3, 4.

## Discussion

This study examined changes in the severity of OCD symptoms in patients with pre-existing OCD diagnoses and specifically with fear of contamination in the state of Qatar during the COVID-19 pandemic. The main finding of this study is that there was worsening of OCD symptoms for individuals with a relatively shorter duration of existing OCD diagnosis (<10 years) during the COVID-19 pandemic compared to pre-pandemic symptom severity.

Published evidence so far has largely looked at the impact of COVID-19 on OCD in general, rather than focusing specifically on OCD with contamination fears or with the duration of the OCD. We set out to study this subset of the affected population as the main strategy for the control of the pandemic has been an emphasis on the prevention of the spread of infection by avoiding close contact and ritualistic hand hygiene measures (20). While a necessary measure, it has resulted in an increase in fears of contamination in the general public and vulnerable populations triggering obsessive-compulsive type symptoms fuelled by fears of contamination (21, 22). Two of the four main symptom domains of OCD are the fear of contamination and washing rituals (29). Amongst the diverse symptom dimensions of OCD, the most frequently recurring themes are obsessions related to contamination (29). There is a growing evidence base that emphasizes the importance of symptom subtypes of OCD in relation to its course, treatment response, and prognosis (30–32). This study was therefore careful in selecting only those participants whose obsessions and compulsions were directly linked to the infection control measures of COVID-19.

We hypothesize that those with a long duration of OCD symptoms were not significantly affected by the COVID-19 infection control measures due to the stability of their symptoms and presumably wider exposure to varying situations. This has an important public health implication of directing extra help and support for those with shorter duration and newly diagnosed OCD. Identifying people at risk of worsening OCD and other mental health problems during this or any future pandemics is important to help formulate informed utilization of limited public health resources. This is in line with the World Health Organization's Policy Brief: COVID-19 and the Need for Action on Mental Health and other global mental health organizations (33).

It is important to note that our data was collected in January 2021, and there is evidence the increase in mental distress that occurred during the emergence of COVID-19 largely diminished in the months to follow due to the population-level resilience in mental health (34). It is possible that as the duration and the nature of the pandemic evolves, these subset differences might disappear.

Given the conflicting evidence arising from the reported studies so far, this study, although exploratory, highlights important caveats that future studies should keep in mind before carrying out population-based studies on COVID-19 in this pandemic. Future studies should consider the subtypes of OCD and also the duration of the diagnosis of OCD. Further, as this pandemic evolves, prospective longer-term studies will help us understand not just the impact of this pandemic on this disorder but will possibly also shed some light on the nature of the disorder itself.

### Limitations

The study design was exploratory, we did not set and formulate any prior statistical hypothesis and did not have a control group. There is a range of potential mechanisms by which COVID-19 could impact OCD and cohort studies are necessary to determine the incidence, etiology, and prognosis of COVID-19-associated OCD.

## Data Availability Statement

The raw data supporting the conclusions of this article will be made available by the authors, without undue reservation.

## Ethics Statement

The studies involving human participants were reviewed and approved by Institutional Review Board, Hamad Medical Corporation. The patients/participants provided their written informed consent to participate in this study.

## Author Contributions

All authors listed have made a substantial, direct, and intellectual contribution to the work and approved it for publication.

## Conflict of Interest

MS, OW, JC, MA, ArA, AbA, EK, PC, and SR were employed by Hamad Medical Corporation.

## Publisher's Note

All claims expressed in this article are solely those of the authors and do not necessarily represent those of their affiliated organizations, or those of the publisher, the editors and the reviewers. Any product that may be evaluated in this article, or claim that may be made by its manufacturer, is not guaranteed or endorsed by the publisher.
